# Providing Nutrition Guidance in the Absence of Conclusive Evidence: What Is a Clinician to Do?

**DOI:** 10.1016/j.jacadv.2026.102670

**Published:** 2026-04-22

**Authors:** Lawerence J. Appel, Alice H. Lichtenstein

**Affiliations:** a Johns Hopkins University School of Medicine, Baltimore, Maryland, USA; b Johns Hopkins Bloomberg School of Public Health, Baltimore, Maryland, USA; c Jean Mayer USDA Human Nutrition Research Center on Aging, Tufts University, Boston, Massachusetts, USA

**Keywords:** counseling, nutrition, prevention

**Figure:**
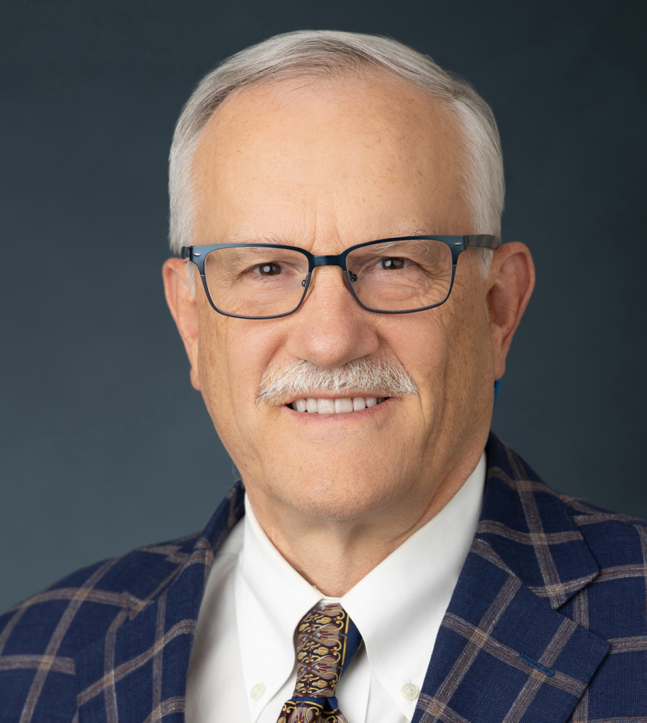

In clinical medicine and public health, decision-making in the setting of inconclusive or sparse evidence is commonplace. Nonetheless, patients as well as the general public seek advice and expect answers from health care providers and public health experts. Guidance on what to eat and drink is commonly requested given the well-founded belief that diet can maintain health by preventing and treating disease. The need for this guidance is of critical importance now, given the tsunami of nutrition information, misinformation, and disinformation that surrounds us. In contrast to other types of diseases, for cardiovascular disease (CVD) and its risk factors, there is a robust body of evidence that links dietary habits to outcomes. Nonetheless, there are evidence gaps, leading to controversies related to the effects of specific foods and nutrients on CVD outcomes.

The paper by Miller et al^1^ in a recent issue of *JACC: Advances* provides an up-to-date, evidence-based discussion of 8 contemporary controversies directly related to diet and CVD risk. Most controversies are well-known to the nutrition community and are often based on a reasonably large body of inconsistent evidence or in some cases strong biological plausibility. However, some of the controversies were unanticipated, such as the health effects of seed oils and beef tallow, which are addressed in their paper.

In general, we concur with their summary of evidence, as well as the assessment of foods and nutrients deemed to be detrimental. Regarding beef tallow, there is no evidence of benefit and considerable evidence of possible harm given its deleterious effects on low-density lipoprotein cholesterol concentrations.^2^ For ultra-processed foods (UPFs), the topic is nuanced. This term includes a large, heterogeneous array of foods and beverages. In general, there is strong evidence that dietary patterns high in UPFs are associated with adverse cardiovascular outcomes.^3^ However, UPFs have very different nutrient profiles, typically one of more of the following: high added sugars, excess sodium, refined carbohydrates, high caloric density, and low nutrient density. However, there are some UPFs that might be considered part of a healthy dietary pattern, such as prepackaged whole grain bread, unsweetened soy milk, and reduced sodium canned beans. This heterogeneity raises a major challenge for clinicians when providing UPF dietary guidance and suggests a need for a more specific set of recommendations rather than a single generic recommendation.

As for artificial sweeteners, a modest but increasing body of evidence from observational studies has associated adverse cardiometabolic consequences with high consumption of artificial sweeteners.^4^ However, the association might be spurious, resulting from reverse causality, that is, persons choosing products containing artificial sweeteners may be more likely to have excess body weight or type 2 diabetes. Furthermore, there is a large number of chemically diverse non-nutritive sweeteners in the marketplace, and with that diversity comes a range of risk associations that are difficult to attribute to specific sweeteners.

Evidence on the benefits of plant oils, including the newly designated subcategory, seed oils, is compelling when they replace sources of saturated fat. The recommendation to eat seafood is without controversy for the general population, with targeted guidance for pregnant women and young children to avoid types of seafood high in mercury.^5^ In contrast, there is ongoing controversy about the role of fish oil supplementation, a topic which was not covered, particularly for primary prevention. As for medium-chain triglycerides, monk fruit, and stevia, available evidence is insufficient to draw any conclusion, leaving the clinician in a difficult position.

Of the 8 controversies discussed by Miller et al^1^, the most contentious is full-fat dairy products. The nutritional value of dairy (bovine) products within a healthy dietary pattern is without controversy. Whether to recommend full-fat or low-fat/fat-free dairy products comes with considerable controversy. Based on the results of a recent review, prospective studies provide little guidance, with the strength of the evidence graded as limited, particularly in terms of cardiovascular risk.^6^ For CVD mortality and blood lipids, the data base was judged as inadequate to draw a conclusion. Prospective cohort studies suggest a more modest effect of dairy than meat fat on low-density lipoprotein cholesterol concentrations.^7^ However, contributing to even a modest effect over a lifetime should not be underestimated.^8^ At this time absent are clear benefits of recommending full fat in place of low-fat/fat-free. Consistent with the prevailing recommendation to consume <10% of energy as saturated fat, it is prudent to recommend low-fat/fat-free options.

In conclusion, the paper by Miller and colleagues provides a well-organized and useful summary of evidence on several diet-related controversies which will likely be raised with health care providers. To provide practical guidance on these controversies, we offer recommendations (Table 1) based on our knowledge of the available literature, often gleaned from serving on committees that developed dietary guidance.

**Table 1:** Recommended Guidance for Cardiovascular and Metabolic Health

Topic	Recommendations
Beef tallow	Avoid beef tallow given its adverse effects on LDL-cholesterol, which is causally related to cardiovascular disease
Ultra-processed foods (UPFs)	In the absence of an implementable definition of UPFs, avoid foods and beverages high in salt, added sugars, and refined carbohydrates, as well as processed meats and ready-to-eat meals.
Artificial sweeteners and sugar alcohols	Reduce overall sweetness preference by prioritizing water, fruits, and unsweetened foods and beverages that are important components of healthy dietary patterns.
Full-fat dairy	When consuming dairy products, choose versions that are low-fat or fat-free instead of full-fat dairy products.
Medium-chain triglycerides	No recommendation^a^
Mock fruit and stevia	No recommendation^a^
Plant (including seed) oils	Use plant oils rather than tropical oils (coconut, palm, and palm kernel) and animal fats (butter, beef tallow, and lard)
Seafood	Consume at least 2 servings per week of seafood, particularly in place of entrees high in saturated fat such as meat and full-fat dairy (eg, quiche).

LDL = Low-density lipoprotein.

a Insufficient evidence to provide guidance on benefit or harm.

## Funding support and author disclosures

Dr Appel served on the 2005 and 2010 Dietary Guidelines Advisory Committees (DGAC) and Dr Lichtenstein served on the 2000 and 2015 DG SAC. Drs Lichtenstein and Appel were Chair and Vice-Chair of the American Heart Association Committee that prepared its 2006 and 2021 scientific statements on dietary guidance to promote cardiovascular health and prevent cardiovascular disease.
